# Salvage with a Secondary Infrahepatic Cavocavostomy of the Occluded Modified Piggyback Anastomosis during Split Liver Transplantation: A Case Report

**DOI:** 10.1155/2014/740802

**Published:** 2014-05-21

**Authors:** Erdem Kinaci, Cuneyt Kayaalp, Sezai Yilmaz, Emrah Otan

**Affiliations:** ^1^Inonu University, Liver Transplantation Institute, 44280 Malatya, Turkey; ^2^Istanbul Training and Research Hospital, Department of General Surgery, 34098 Istanbul, Turkey

## Abstract

Hepatic venous outflow obstruction following liver transplantation is rare but disastrous. Here we described a 14-year-old boy who underwent a split right lobe liver transplantation with modified (side-to-side) piggyback technique which resulted in hepatic venous outflow obstruction. When the liver graft was lifted up, the outflow drainage returned to normal but when it was placed back into the abdomen, the outflow obstruction recurred. Because reanastomosis would have resulted in hepatic reischemia, alternatively, a second infrahepatic cavocavostomy was planned without requiring hepatic reischemia. During this procedure, the first assistant hung the liver up to provide sufficient outflow and the portal inflow of the graft continued as well. We only clamped the recipient's infrahepatic vena cava and the caudal cuff of the graft cava. After the second end-to-side cavocaval anastomosis, the graft was placed in its orthotopic position and there was no outflow problem anymore. The patient tolerated the procedure well and there were no problems after three months of follow-up. A second cavocavostomy can provide an extra bypass for some hepatic venous outflow problems after piggyback anastomosis by avoiding hepatic reischemia.

## 1. Introduction

There are several vascular complications of liver transplantation diagnosed intraoperatively. Venous outflow obstruction during liver transplantation is a serious problem which may lead to loss of the graft. It can be corrected with a reanastomosis; however, this is potentially harmful due to the necessity of reclamping of hepatic inflow that prolongs the warm ischemia time. Here, a case of hepatic outflow obstruction during a split right lobe liver transplantation with a modified piggyback cavocaval anastomosis technique was described. The problem was solved by an infrahepatic second cavocaval anastomosis without occlusion of the hepatic inflow or outflow.

## 2. Case Presentation

A 14-year-old boy, weighing 24 kg, with hepatic failure secondary to Wilson's disease was admitted for liver transplantation. Physical examination revealed decreased skin tonus, jaundice, malnutrition, oliguria, and depression of mental status. Among laboratory test results, aspartate aminotransferase was 251 IU/L, alanine aminotransferase was 121 IU/L, alkaline phosphatase was 267 IU/L, gamma glutamyl transpeptidase was 56 IU/L, creatinine was 0.7 mg/dL, albumin was 4.1 g/dL, total bilirubin was 31.8 mg/dL, and INR was 1.6. Child-Pugh score was 8 and Model for End-Stage Liver Disease (MELD) score was 25.

During the transplant waiting period, a deceased liver was accepted from the National Organ Sharing System for a small baby who had acute liver failure. The full-size liver was divided into two parts as the left lobe was for this small baby and the remaining right lobe with the caudate lobe and retrohepatic inferior vena cava was used for the 14-year-old patient. The graft belonged to a 31-year-old male, weighing 60 kg. The right lobe graft weighed 695 g and the graft to recipient weight ratio (GRWR) was 2.89%.

At the back table, the graft was prepared for the modified piggyback cavocaval anastomosis. Supra- and infrahepatic orifices of the graft vena cava were closed with 5/0 polypropylene sutures. It was anastomosed to the recipient's vena cava by side-to-side, under total caval clamping. A 4 cm length anastomosis was performed to the most cranial parts of the recipient's and the donor's vena cava. Anastomosis was accomplished to the anterior wall of the recipient vena cava without removing any slit from the cava. Before the portal anastomosis, preservation solution in the liver was washed out through a small orifice at the corner of the donor's infrahepatic vena cava and this orifice was closed by sutures after wash-out. Portal vein anastomosis was completed and reperfusion was accomplished.

**Figure 1:**
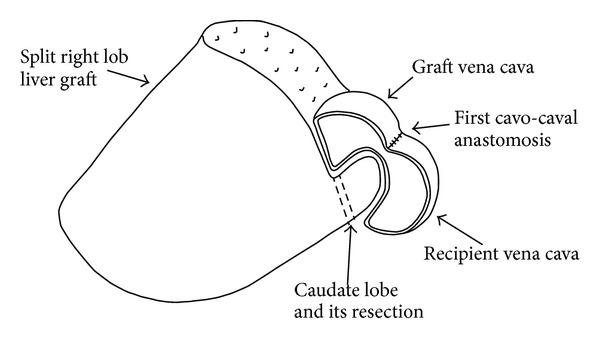
The caudate lobe was resected.

**Figure 2:**
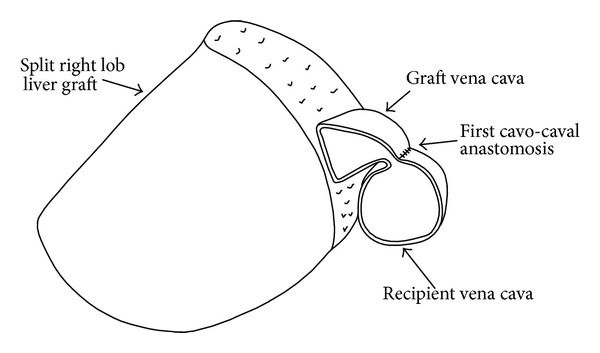
Rotational effect on the cavocaval anastomosis.

The systolic arterial blood pressure was between 70 and 90 mmHg before the implantation and it dropped to 40 mm/Hg following reperfusion and did not recover. The liver became hard and dark, and venous bleeding came out from the cut surface. When we lifted the graft up, blood pressure increased to normal level, the tonus/color of the liver returned to normal, and cut surface bleeding ceased. When we left the graft to the right subphrenic space back, the same bad scenario repeated. At first, to eliminate the possibility of positional occlusion, we filled the right subphrenic space with a compress; however, the liver was not released. Secondly, to exclude the external pressure on the inferior vena cava, we resected the caudate lobe of the graft, but it did not provide any benefit (Figure 1). During all those maneuvers, the liver was always hung up by the first assistant. Afterwards, we checked the anastomosis externally by a finger through the recipient vena cava. There was no mechanical occlusion at the anastomosis, when the liver was hung up. However, we believed that a positional kinking of the cavocaval anastomosis could be the reason due to the anterior caval wall anastomosis without removing any caval slit (Figure 2). A reconstruction of the caval anastomosis was planned, but it would be risky because of the necessity of reclamping of the hepatic inflow and outflow that would result in a second ischemia/reperfusion injury of the split graft. Instead, a second anastomosis between both cavae without the risk of an additional second hepatic ischemia/reperfusion injury was preferred.

**Figure 3:**
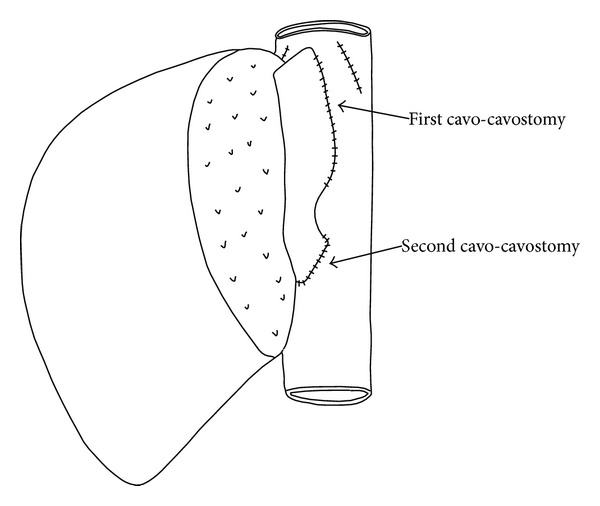
The second cavocaval anastomosis.

Caudal stump of the donor's vena cava clamped and the sutured end was released. The infrahepatic vena cava of the recipient was totally cross-clamped. The graft was lifted up for good drainage during the anastomosis. We performed an infrahepatic anastomosis between the end of the graft's vena cava and the anterolateral wall of the recipient's vena cava. During the procedure, the venous inflow and outflow of the graft were not interrupted. After the new cavocavostomy, the caval clamps were released and the graft was left in the right subphrenic space (Figure 3). Systolic arterial pressure was normal and the graft seemed soft, normal in color, and without bleeding. Finally, the reconstruction of the hepatic artery and bile duct was completed. The postoperative course was uneventful and the patient was alive on the 3rd month of the transplantation.

## 3. Discussion

Several mechanisms can cause hepatic venous obstruction after liver transplantation. Positional problems can be a reason and placement of tissue expanders behind the graft is an option for correction [1] and compression of the native infrahepatic caval vein has also been described before [2]. External compression of the caudate lobe could be another defined mechanism for caval flow obstruction [3]. Risaliti [4] reported that venous outflow obstruction can be the result of the size discrepancy between the donor's suprahepatic vena cava and the stump of the recipient's hepatic veins. In this case, the GRWR was 2.89% and we checked the piggyback anastomosis but the obstruction was not related to size discrepancy. Outflow problems during and after liver transplantation are more common with reduced-size livers and the reason is usually the kinking of the venous anastomosis due to the rotation effect of a relatively small graft [5]. Large grafts also can be a reason of positional outflow obstruction [6]. In our case, a positional obstruction (rotational effect) at the anastomosis was thought to be the reason for the outflow problem. The patient's primary diagnosis was Wilson's disease and the removed liver was bulky with a deep subphrenic space. Despite the large volume of the graft, there should be a rotation effect. Possibly, anterior located side-to-side anastomosis without slit removal aggravated the obstructive problem. The second anastomosis (cavocavostomy) was located anterolaterally to the recipient's cava and in an end-to-side manner. At the end, the hepatic outflow was provided successfully.

A similar secondary caval anastomosis during liver transplantation was described in the literature by partial side clamping of the recipient's cava [4, 7]. In those cases, the piggyback techniques included the right and left hepatic vein orifices. Therefore, they had enough caval length for the secondary anastomosis. In our case, partial side clamping was not easy for the upper long anastomosis. Therefore, a total infrahepatic caval clamping and end-to-side anastomosis were preferred. It took almost 15 minutes without any serious hemodynamic instability. Quintini and coworkers [8] described a side-to-side cavocavostomy with an endovascular stapler to rescue the obstructed hepatic outflows in two deceased full-size liver grafts. Their cases were done under total hepatic vascular clamping; in other words, they clamped the hepatic artery and portal vein as well. We believe that performing an extra anastomosis without any additional hepatic ischemia is one of the major advantages of our technique.

Regardless of the reason, hepatic venous outflow occlusion and its treatment by an additional anastomosis after piggyback liver transplantation have been described in a limited number of studies [4, 6–8]. This paper presented the first case of a second anastomosis for outflow occlusion after a split liver transplantation. Moreover, this was the first case of a pediatric patient related to this topic.

As a conclusion, an infrahepatic second cavocavostomy is a good option for the intraoperative management of some hepatic venous outflow obstruction without resulting in any additional graft ischemia.
